# Cross-Cultural Nutritional Epigenomics: Diet and Microbiome Interactions Shaping Type 2 Diabetes in Arab and Western Populations

**DOI:** 10.3390/nu18040681

**Published:** 2026-02-20

**Authors:** Tarek Arabi, Arshiya Akbar, Ahmed Yaqinuddin, Mohammed Imran Khan, Itika Arora

**Affiliations:** 1 College of Medicine, Alfaisal University, Riyadh 11533, Saudi Arabia; tarabi@alfaisal.edu (T.A.); arshiyaakbar2019@gmail.com (A.A.); mikhan@kfshrc.edu.sa (M.I.K.); 2 King Faisal Specialist Hospital and Research Centre, Jeddah 21499, Saudi Arabia; 3 Biotechnology Centre, Khalifa University, Abu Dhabi 127788, United Arab Emirates

**Keywords:** diet, gut microbiome, epigenome, DNA methylation, histone modifications, non-coding RNAs, type 2 diabetes, Arab/MENA, Western cohorts

## Abstract

In the Middle East and North Africa (MENA) region, the prevalence of Type 2 Diabetes (T2D) is 17–18%, substantially higher than the ~9–10% reported in Western populations, with some Gulf states approaching 25% in adults. Historically, Arab diets, characterized by high fiber intake from whole grains, legumes, and fermented dairy products, have contrasted markedly with the Western dietary pattern increasingly prevalent among urbanized Arab populations. These nutritional shifts have been associated with changes in gut microbial composition, including lower representation of short-chain fatty acid–producing bacteria and higher abundance of dysbiosis-associated taxa. Concurrently, diet-derived compounds and microbial metabolites have been associated with changes in DNA methylation, histone modifications, and non-coding RNA expression. Epigenome-wide association studies revealed both shared and population-specific methylation signatures in patients with T2D. However, integrated multi-omics studies remain limited in Arab populations, where the disease burden is highest. This review integrates emerging evidence on diet-linked epigenetic alterations, microbiome-associated metabolic pathways, and their intersection in potentially contributing to T2D risk and progression. Given the heterogeneity of T2D across populations, there is a pressing need for culturally contextualized precision medicine frameworks that integrate population-specific diet–microbiome–epigenome dynamics rather than extrapolating findings across populations. Additionally, this review synthesizes evidence that dietary patterns are associated with T2D-relevant pathways through the diet–microbiome–epigenome axis, with emphasis on Arab/MENA populations and Western comparator cohorts.

## 1. Introduction

Type 2 Diabetes (T2D) has reached pandemic proportions worldwide, with an especially severe impact in Arab populations compared to Western countries [1]. In the Middle East and North Africa (MENA) region, nearly one in six adults (approximately 17–18 percent of the population) is living with diabetes, roughly double the prevalence observed in Europe (around 9–10 percent) [1]. Rapid nutritional transitions, urbanization, and sedentary lifestyles accompanying economic development have substantially increased obesity and metabolic disease in Arab societies [2]. Previous studies have demonstrated that Arab populations exhibit increased susceptibility to T2D when exposed to environmental stressors [3]. Amid this escalating burden, increasing evidence indicates that the heterogeneity of T2D across populations can be more comprehensively understood through the interplay between diet, the epigenome, and the gut microbiome. Dietary patterns shape gut microbial composition and function and induce epigenetic modifications, which collectively modulate metabolic homeostasis and disease susceptibility [4]. This integrative model of diet–microbiome–epigenome interactions provide a compelling lens for understanding the divergent T2D prevalence and metabolic phenotypes observed between Arab and Western populations. Traditional Arab and Western dietary patterns differ substantially, and these differences may contribute to distinct gut microbial configurations and epigenomic landscapes that influence glucose homeostasis.

In this structured scoping review with narrative synthesis, we examine how dietary exposures influence epigenetic and microbial determinants of T2D across Arab and Western populations. We first outline the epidemiological landscape, followed by a comparative analysis of dietary patterns characteristic of these regions. We then synthesize current evidence on diet-driven epigenetic regulation and gut microbiome alterations and their roles in shaping metabolic phenotypes relevant to T2D. Particular emphasis is placed on the bidirectional interactions among diet, microbiota, and the epigenome, and on their implications for advancing precision nutrition and precision endocrinology in diverse populations.

Rather than treating Arab and Western populations as homogeneous or mutually exclusive categories, we use them as heuristic reference points to illustrate how differences in dietary transition, food environments, and sociocultural contexts modulate diet–microbiome–epigenome interactions relevant to T2D risk. We argue that T2D is a biologically heterogeneous disorder whose pathogenesis and therapeutic optimization depend on culturally and environmentally specific diet–microbiome–epigenome interactions. Precision-medicine frameworks must therefore incorporate these population-specific architectures, rather than extrapolating from Western-centric models that inadequately represent heterogeneity across North American and European food systems.

### Literature Search Strategy and Study Selection

We conducted a structured scoping review with narrative synthesis to identify evidence on cross-cultural associations among diet, gut microbiome composition and function, epigenetic regulation, and T2D, with a focus on Arab/MENA and Western populations. PubMed/MEDLINE, Scopus, and Web of Science were searched for studies published between January 2000 and March 2025. Search strategies combined controlled vocabulary and free-text terms related to: (i) T2D (“T2D”, “T2DM”, “insulin resistance”); (ii) diet (“dietary pattern”, “Western diet”, “Mediterranean diet”, “fiber”, “polyphenols”, “fermented foods”); (iii) the microbiome (“gut microbiome”, “metagenomics”, “16S rRNA”, “shotgun sequencing”, “short-chain fatty acids”); (iv) epigenetics (“DNA methylation”, “histone modification”, “microRNA”, “epigenome-wide association”); and (v) population context (“Arab”, “Middle East”, “MENA”, “Gulf”, “Europe”, “North America”). Reference lists of key reviews and eligible primary studies were also screened to identify additional articles.

We prioritized human observational studies, randomized or quasi-experimental dietary and lifestyle intervention trials, and population-based epigenetic studies (including EWAS and candidate-locus analyses) reporting outcomes related to glycemia, insulin sensitivity, inflammatory markers, microbiome composition or function, and epigenetic modifications. Mechanistic evidence from in vitro or animal models was included to support biological plausibility when human causal inference was limited. We excluded studies that were not focused on T2D-related metabolic outcomes, investigated non-gut microbiomes, or lacked sufficient methodological detail. Owing to heterogeneity in study design, population characteristics, and analytical platforms, findings were synthesized narratively, with explicit attention to confounding, methodological limitations, and the distinction between association and causation.

## 2. Global and Regional Epidemiology of T2D

The global prevalence of diabetes has risen substantially over recent decades and is projected to continue increasing. In 2019, an estimated 463 million adults (approximately 9.3% of the global adult population) were living with diabetes [5]. Projections from the International Diabetes Federation estimate that this number will reach 783 million by 2045 [1], while Global Burden of Disease analyses suggest that the total burden could exceed one billion by mid-century under current trajectories [6,7]. This rise exceeds what would be expected from population growth alone. It has been attributed largely to deteriorating diet quality, increasing adiposity, and declining physical activity, positioning T2D as a major long-term public health challenge.

Global diabetes prevalence is not uniformly distributed, but exhibits pronounced geographic disparities. The Middle East and North Africa (MENA) region reports among the highest prevalence rates worldwide, affecting approximately 17–18% of adults [8]. Within MENA, Gulf countries such as Kuwait, Qatar, and Saudi Arabia consistently rank among the highest globally; for instance, Kuwait’s adult diabetes prevalence is estimated at approximately 25–26% [1,8]. This burden is compounded by high obesity prevalence, sedentary lifestyles, and rapid nutritional transition associated with urbanization and economic growth [9]. For comparison, adult diabetes prevalence in many Western settings is lower (approximately 11–14% in the United States and 7–10% across parts of Europe), underscoring the disproportionate metabolic burden borne by Gulf Arab populations [8,10]. These regional inequities highlight the need for population-specific investigation of diet–microbiome–epigenetic interactions in T2D, rather than assuming direct transferability of mechanistic insights derived predominantly from Western cohorts [6].

## 3. Demographic and Socioeconomic Patterns

Beyond geographic variation, T2D exhibits consistent demographic gradients across populations. One well-documented pattern is the urban–rural disparity, with higher prevalence observed in urban settings. This gradient likely reflects greater exposure to energy-dense, ultra-processed foods, reduced occupational and incidental physical activity, and more sedentary lifestyles [5]. The urban predominance of T2D is evident in both Arab and Western contexts and may be particularly pronounced in rapidly urbanizing Middle Eastern societies.

Age at onset represents another clinically significant distinction. In Western Europe and North America, T2D has historically been diagnosed predominantly in midlife, whereas in several Arab populations, the onset occurs at younger ages, frequently in the third to fourth decades of life. Earlier onset extends cumulative exposure to hyperglycemia and increases the lifetime risk of microvascular and macrovascular complications [10,11]. Socioeconomic status (SES) further modifies risk. However, its distribution varies by context: in many high-income Western settings, T2D disproportionately affects lower-SES groups, whereas in MENA the burden spans both affluent urban populations and underserved communities, reflecting rapid lifestyle transitions, nutritional shifts, and disparities in preventive care and health literacy [5].

### 3.1. Physical Activity as a Modulator of Insulin Sensitivity, Microbiome, and Epigenetic Marks

Physical activity is a well-established and clinically significant determinant of insulin sensitivity, systemic inflammation, and T2D risk [2]. Beyond its metabolic effects, it modulates gut microbiome composition and epigenetic regulation in key metabolic tissues, intersecting with the diet–microbiome–epigenome axis [4]. Regular exercise is associated with enhanced gut barrier integrity and reduced pro-inflammatory signaling. Human studies report shifts toward a more favorable microbial profile, including increased abundance of short-chain fatty acid (SCFA)-producing taxa in some cohorts. Concurrently, physical activity induces epigenetic remodeling in skeletal muscle, liver, and adipose tissue, including alterations in DNA methylation and histone modifications at loci involved in glucose transport, mitochondrial biogenesis, and inflammatory pathways. These adaptations provide a mechanistic foundation for sustained improvements in metabolic homeostasis.

These considerations are particularly relevant in cross-cultural comparisons because physical activity patterns differ substantially across many Arab and Western settings, owing to urbanization, climate, transportation norms, and sociocultural factors, including gender-related barriers and sedentary occupations [2]. Therefore, physical activity should be treated as a key confounder and effect modifier when interpreting associations among diet, microbiome composition, and epigenetic signatures in T2D, especially in observational cohort studies.

### 3.2. Key Confounders and Effect Modifiers in Diet–Microbiome–Epigenome Studies

Interpretation of diet–microbiome–epigenome associations in T2D requires explicit consideration of confounders and effect modifiers that vary across populations. Accordingly, cross-regional differences in diet, microbiome composition, and metabolic outcomes may reflect not only dietary patterns per se, but also variation in physical activity, medication use, socioeconomic context, and population genetic structure [2].

Physical activity patterns differ substantially between many Arab and Western contexts, reflecting urbanization, climatic constraints, and sociocultural factors, including gender-related barriers [2]. Medication use, particularly metformin, is a well-established modifier of gut microbiome composition and may also influence epigenetic profiles. Population genetic structure, including admixture and consanguinity patterns characteristic of some Arab populations, may further shape baseline microbiome configurations and epigenetic variation, thereby limiting the direct transferability of biomarker models across populations [3].

For these reasons, cross-cultural comparisons of diet–microbiome–epigenome interactions should prioritize harmonized metadata collection (including dietary assessment instruments, medication history, physical activity patterns, and population ancestry) and analytic strategies that explicitly account for these contextual factors.

## 4. Early-Onset and Prediabetes

One of the most concerning epidemiological shifts has been the marked rise in early-onset T2D. Once considered rare in youth, T2D is now increasingly diagnosed in adolescents and young adults, particularly in Gulf countries. This trend reflects profound changes in dietary patterns, physical activity, and overall metabolic health, with important implications for cumulative glycemic exposure and long-term complications [11]. Pediatric endocrinology reports from Saudi Arabia, Kuwait, and Qatar document T2D diagnoses in adolescents as young as 13 years, frequently associated with severe childhood obesity and strong family histories of diabetes [12,13,14]. Consequently, a substantial proportion of individuals in their third and fourth decades of life in these settings already exhibit overt diabetes or impaired glucose regulation.

Prediabetes, a precursor state of dysglycemia, is also highly prevalent in the MENA region. Approximately 7% of adults are estimated to have impaired glucose tolerance (IGT), one of the principal clinical forms of prediabetes [15]. Several MENA countries report relatively rapid progression from prediabetes to overt diabetes, consistent with a high-risk metabolic environment characterized by obesity, physical inactivity, and dietary transition. In some Gulf clinical settings, the mean age at prediabetes diagnosis has been reported in the third decade of life [16]. The combination of earlier onset and a large reservoir of individuals with prediabetes have important implications for future diabetes incidence, complication rates, and healthcare expenditures. These trends underscore the need for population-level screening strategies and targeted preventive interventions to delay or prevent progression to overt T2D [11].

## 5. Burden Metrics

T2D imposes a substantial global health and economic burden. In 2021, diabetes was estimated to account for approximately 6.7 million deaths worldwide, largely attributable to cardiovascular complications, and ranked among the leading contributors to disability-adjusted life years (DALYs) across multiple regions [17,18]. Global healthcare expenditure on diabetes reached an estimated USD 966 billion in 2021 and is projected to surpass USD 1 trillion annually in the coming decades [1]. A pronounced resource imbalance is evident in high-burden regions: the MENA region accounts for a substantial proportion of global diabetes cases but a disproportionately smaller share of diabetes-related healthcare spending, contributing to inequities in access to optimal care and adverse clinical outcomes [8,19].

## 6. Data Gaps and Surveillance Challenges

Accurate epidemiological data are essential for effective T2D surveillance, prevention, and policy planning; however, substantial gaps remain, particularly across the Arab world. Many MENA countries lack comprehensive, nationally representative diabetes surveillance systems and rely on extrapolated estimates or intermittent population-based studies. In 2019, the International Diabetes Federation (IDF) derived prevalence estimates for several Arab countries using modeling approaches due to the absence of recent national survey data [9]. Heterogeneity in study design, sampling frameworks, and diagnostic criteria further complicates cross-country comparisons. Consequently, reported prevalence figures may underestimate the true burden of disease.

For example, an estimated one in three adults living with diabetes in the MENA region is undiagnosed and silently developing complications [8]. High levels of underdiagnosis, coupled with inconsistent screening practices, indicate that reported prevalence figures likely capture only a fraction of the true disease burden. In many rural areas and conflict-affected countries in the Middle East, systematic surveillance data for T2D remain limited or absent. Therefore, there is a pressing need for standardized, population-based registries and regular screening programs across the region [20]. As a result, improved data collection would support more effective resource allocation and enable more accurate evaluation of intervention outcomes. Encouragingly, several Gulf states, including Qatar and the United Arab Emirates, have established diabetes registries in recent years. However, coverage remains incomplete and inconsistent across countries.

In summary, while the broad trajectory of the T2D epidemic in the MENA region is well recognized, the finer epidemiological details remain uncertain due to data limitations. Strengthening surveillance systems across the region is therefore essential to inform public health policy and accurately track progress.

## 7. Dietary Patterns in Arab and Western Populations

Diet is a fundamental determinant of metabolic health, and dietary patterns vary between traditional Arab cuisines and the modern Western nutritional model. Historically, Arab diets were predominantly plant-based and high in fiber, emphasizing balance and moderation. Everyday staples included whole grains such as bulgur and whole-wheat flatbread; a variety of legumes, such as lentils and chickpeas (featured in dishes such as falafel and ful medames); and abundant vegetables and fruits, particularly dates, which served as natural sources of energy and essential nutrients [21]. Fermented dairy products, such as laban and labneh, were also traditional staples, providing protein, calcium, and probiotics that support gut health and overall nutrition.

Certain traditional dietary patterns in parts of the MENA region share features with Mediterranean-style diets, characterized by high intakes of fiber, complex carbohydrates, and healthy fats such as olive oil, while remaining low in refined sugars. Such dietary habits have been consistently linked to a lower risk of obesity and diabetes, with evidence from multiple studies showing that diets rich in fruits, vegetables, legumes, and whole foods provide strong protection against metabolic diseases [22]. However, dietary practices across the MENA region are heterogeneous and not uniformly protective, and metabolic risk depends on overall dietary quality, portion size, and lifestyle context.

The term “traditional Arab diet” does not denote a single, uniform dietary pattern across the MENA region. Dietary practices vary substantially by country, rural versus urban setting, socioeconomic context, and historical period, including intervals of food scarcity and reliance on energy-dense staples. Furthermore, traditional dietary practices are not inherently protective; cardiometabolic risk depends on overall dietary quality, portion size, and broader lifestyle context. Herein, we use the term “traditional” as a descriptive reference point rather than as a normative or uniformly health-promoting standard.

The Western dietary pattern, now widely disseminated globally, is characterized by high intake of ultra-processed foods, red and processed meats, saturated fats, and refined carbohydrates, including white flour and sugar-sweetened beverages. Over the past three to four decades, many Arab countries have undergone a pronounced nutritional transition toward Westernized dietary practices [23]. Rapid urbanization, particularly in oil-rich Gulf states, and globalized food systems have contributed to increased consumption of fast food, packaged snacks, and sugar-sweetened beverages across the Middle East. National dietary data indicate substantial increases in per capita sugar and fat intake in Gulf countries from the 1980s through the 2010s, accompanied by a decline in dietary fiber intake [21,24]. In many settings, energy-dense convenience foods have progressively displaced traditional home-prepared meals. Sociocultural shifts, including the expansion of large commercial retail spaces and international food franchises, have further reshaped food environments in metropolitan centers such as Riyadh and Dubai. Concurrently, portion sizes and total caloric intake have increased, partly due to hospitality norms that emphasize the provision of abundant food at social gatherings [24].

Although “Western diet” is often used as a single label, dietary patterns and food environments differ substantially across Western settings. North American cohorts are frequently characterized by higher exposure to ultra-processed foods and sugar-sweetened beverages. In contrast, European cohorts vary widely and may include Mediterranean, Nordic, or other traditional dietary patterns with distinct microbiome and metabolic associations. Accordingly, where Western comparator data are discussed, we interpret them as context-dependent rather than as a uniform reference category. These dietary shifts are associated with measurable differences in gut microbiome profiles and metabolic risk markers in multiple studies. High-fiber diets typical of traditional Arab eating patterns support a diverse gut microbiota enriched with beneficial fermenters. Colonic bacteria metabolize fiber from whole grains and legumes into short-chain fatty acids (SCFAs), such as butyrate, which improve insulin sensitivity [25]. Regular intake of fermented dairy products also introduces probiotic organisms. In contrast, a Western diet low in fiber and high in animal fat and sugar promotes a less diverse microbiome and the proliferation of taxa associated with inflammation and insulin resistance [26]. Studies of Western cohorts with T2D have demonstrated depletion of SCFA-producing genera, such as *Faecalibacterium* and *Roseburia*, along with an increased abundance of pathobionts [27]. Processed foods typically lack prebiotic fibers, which are essential for maintaining microbial diversity, and excessive fat and simple sugars can drive gut dysbiosis and metabolic endotoxemia. As dietary patterns in Gulf and wider Arab countries undergo ongoing transition toward more Westernized consumption profiles [21,23,24], parallel increases in obesity and T2D have been widely documented across the region [9,10,11,15,20]. Evidence from experimental and observational studies links Western dietary patterns to gut dysbiosis and metabolic risk [4,26,27], including reduced representation of short-chain fatty acid (SCFA)-producing taxa and altered microbial metabolic pathways [25,26]. While these mechanisms provide a plausible framework for understanding diet–microbiome–metabolic interactions in Arab populations, reported patterns vary across settings, and direct, harmonized microbiome comparisons between Arab and Western cohorts remain limited. Moreover, traditional Arab diets include distinctive components, such as dates, which are rich in polyphenols and soluble fiber, as well as various spices and herbal teas, which may exert protective epigenetic and anti-inflammatory effects. The growing replacement of such foods with ultra-processed products contributes to the loss of these natural metabolic safeguards.

In summary, the Arab world is experiencing a dietary duality: older generations continue to consume traditional, high-fiber diets, while younger generations increasingly adopt Western nutritional patterns. This dietary contrast provides an opportunity to examine how diet influences the pathogenesis of T2D. The Western diet is known to contribute to obesity and diabetes, whereas elements of the traditional Arab/Mediterranean diet, such as olive oil, legumes, and fermented foods, are considered protective [22]. The subsequent sections of this review examine how these dietary differences are reflected in the epigenome and gut microbiota, and how such biological changes may help explain the disproportionately high burden of T2D in Arab populations.

## 8. Dietary Impact on the Epigenome

### 8.1. Epigenetic Mechanisms in T2D

**Figure 1:**
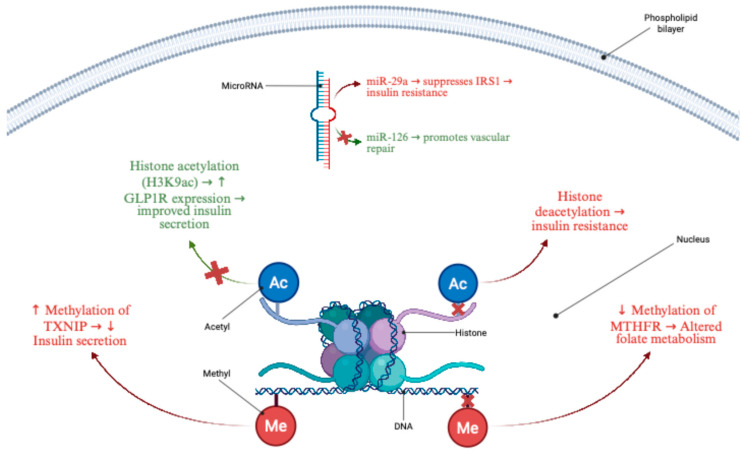
Schematic representation of key epigenetic mechanisms affecting insulin secretion and metabolic homeostasis. The diagram illustrates the three major epigenetic layers within metabolic cells. (1) Blood-based EWAS have reported altered methylation at loci such as TXNIP; however, the functional direction and tissue-specific relevance to pancreatic β-cell biology remain to be fully established. In contrast, reduced methylation of MTHFR alters folate-dependent one-carbon metabolism. (2) Histone modifications: Increased H3K9 acetylation enhances GLP1R expression and improves incretin-stimulated insulin secretion, while elevated histone deacetylation is associated with insulin resistance. (3) MicroRNAs: miR-29a suppresses IRS1, impairing insulin signaling, whereas miR-126 promotes endothelial repair and vascular health. Together, these epigenetic mechanisms converge to modulate β-cell function, insulin sensitivity, and inflammatory tone in T2D.

Epigenetics, referring to heritable changes in gene expression that do not involve alterations in the DNA sequence, plays a central role in the development of T2D (Figure 1). Chronic metabolic stress can trigger epigenetic changes in key metabolic tissues, including pancreatic islets, liver, muscle, and adipose tissue. These modifications alter the expression of genes involved in insulin secretion, insulin signaling, and inflammatory pathways, ultimately influencing glucose metabolism and the progression of T2D [28].

The best-studied epigenetic mechanism is DNA methylation. In patients with T2D, epigenome-wide association studies (EWAS) have identified differential methylation at multiple loci associated with glucose metabolism. For example, epigenome-wide association studies have consistently reported altered *TXNIP* methylation in peripheral blood, which correlates with glycemic traits and is widely used as a biomarker of metabolic stress in T2D [29]. *TXNIP* is typically upregulated by high glucose and can trigger β-cell apoptosis; epigenetic silencing of *TXNIP* appears protective, whereas demethylation and overexpression are observed in diabetes [30,31]. Another locus, *MTHFR*, which is involved in one-carbon metabolism, exhibits methylation changes that may affect the homocysteine and folate pathways in diabetes [32]. Similarly, *NT5C2*, a gene related to purine metabolism, has emerged in several EWAS as differentially methylated in T2D, although its functional significance remains unclear [33].

Beyond DNA methylation, histone modifications also shape gene expression by regulating chromatin accessibility. Acetylation and methylation of histone tails dynamically respond to metabolic signals. In insulin-resistant states, specific histone modifications enhance the expression of inflammatory mediators in adipose tissue and the liver, promoting chronic low-grade inflammation and exacerbating metabolic dysfunction [34]. Epigenetic regulation of *GLP1R*, including increased chromatin accessibility via histone acetylation or inhibition of histone deacetylases (HDACs), have been demonstrated in experimental β-cell models. These changes enhance *GLP-1* receptor expression. This upregulation improves cellular responsiveness to incretin hormones and supports more effective insulin secretion [35,36].

A third layer of epigenetic regulation involves non-coding RNAs. MicroRNAs (miRNAs) and long non-coding RNAs (lncRNAs) fine-tune gene expression at the post-transcriptional and transcriptional levels. For example, hsa-miR-200c is elevated in specific T2D models and can disrupt insulin signaling by targeting IRS-1 and related components; it also influences epithelial-to-mesenchymal transition, potentially contributing to islet cell dysfunction [37,38,39]. Several studies have shown that islet-enriched miRNAs are downregulated in T2D, thereby derepressing target genes that impair insulin secretion [40]. Long non-coding RNAs such as *SNHG16* and *TMEM9B-AS1* have also been implicated in glucose homeostasis [41,42]. *SNHG16* can modulate inflammatory pathways in endothelial cells and is upregulated in diabetes, suggesting an epigenetically driven inflammatory loop that contributes to vascular dysfunction [43,44]. The major epigenetic layers implicated in metabolic regulation and T2D, including DNA methylation, histone modifications, and non-coding RNAs, are schematically illustrated in Figure 1.

Collectively, these epigenetic alterations modulate transcriptional programs regulating β-cell function, insulin signaling, and inflammatory pathways. Such molecular perturbations contribute to core pathophysiological features of T2D, including β-cell dysfunction, insulin resistance, and chronic low-grade inflammation. Unlike fixed genetic mutations, epigenetic modifications are dynamic and responsive to environmental exposures, including dietary factors. This biological plasticity supports the premise that targeted nutritional or pharmacological strategies may partially recalibrate the epigenome toward improved metabolic regulation in individuals at elevated risk for diabetes. Key epigenome-wide and candidate-gene DNA methylation studies relevant to T2D in Arab and comparator populations are summarized in Table 1.

### 8.2. Nutrients and Compounds Affecting Epigenetic Marks

Diet serves as a significant source of epigenetic modulators, as nutrients and bioactive food components can directly influence DNA and histone modifications or regulate the activity and expression of epigenetic enzymes [54,55]. One of the clearest examples involves folate and other methyl donors. Diets rich in folate, vitamin B12, choline, and betaine influence DNA methylation through the one-carbon cycle [56]. Deficiencies in these nutrients or the presence of genetic polymorphisms such as MTHFR C677T can lead to abnormal DNA methylation patterns associated with insulin resistance and impaired glucose metabolism [56,57].

Beyond vitamins, a variety of dietary phytochemicals exert epigenetic effects. Polyphenols such as resveratrol (found in grapes and red wine) and curcumin (derived from turmeric) can modulate the activity of histone acetyltransferases and deacetylases, influencing gene expression in metabolic pathways relevant to glucose regulation and insulin sensitivity [58,59]. Resveratrol has also been shown to activate SIRT1, a histone deacetylase that enhances insulin sensitivity and supports mitochondrial function. Through this mechanism, resveratrol improves metabolic efficiency and protects against insulin resistance [60]. Diets rich in polyphenols, including berries, green tea, and olive oil, are associated with beneficial epigenetic profiles in metabolic tissues, including increased histone acetylation at anti-inflammatory gene loci [61,62].

Dietary fiber can influence epigenetic regulation indirectly via microbiome-derived SCFAs; mechanistic details are discussed in Section 11 and Section 12 [63,64]. In contrast, harmful dietary components can induce epigenetic modifications. For instance, endocrine-disrupting chemicals commonly found in processed foods and packaging materials, such as bisphenol A (BPA) and certain phthalates, have been shown in experimental models to cause DNA hypomethylation at key metabolic genes, promoting adipogenesis and insulin resistance [65,66]. These findings highlight the potential for environmental exposures to influence the risk of metabolic disease through epigenetic mechanisms. Similarly, chronic exposure to dietary advanced glycation end products (AGEs) or certain paraben preservatives may drive pro-diabetic epigenetic modifications, although human data remain limited [67,68].

These “nutri-epigenomic” effects extend to pharmacotherapy as well. The antidiabetic drug metformin exerts epigenetic influences, including induction of miRNAs that enhance insulin signaling and modification of DNA methylation in hepatic gluconeogenic genes [69]. Notably, nutritional status may even predict therapeutic response through epigenetic signatures. The CORDIOPREV trial in Spain demonstrated that baseline circulating microRNA profiles could predict remission of T2D following intensive lifestyle intervention. Patients with higher baseline levels of hsa-miR-141-5p, hsa-miR-182, and hsa-miR-192 were more likely to achieve remission [70]. These findings highlight the potential of miRNA-based biomarkers to guide personalized nutrition and metabolic therapy.

In summary, nutrients and food-derived compounds can act as double-edged swords: polyphenols, SCFAs, and methyl donors induce protective epigenetic changes, whereas processed food chemicals, dietary pollutants, and excessive sugars and fats may instigate harmful epigenetic alterations that promote the development of T2D. Key dietary components, their associated epigenetic mechanisms, and supporting human evidence relevant to metabolic regulation and T2D risk are summarized in Table 2.

## 9. Western vs. Eastern Diets and Epigenetic Regulation

Interventional studies in Western populations, particularly Mediterranean diet trials, have reported associations between dietary adherence and differential DNA methylation at genes involved in glucose metabolism and inflammatory regulation [85,86]. In selected trials, dietary modification has been associated with methylation changes at loci such as TXNIP and adiponectin-related genes, as well as shifts in circulating microRNA profiles relevant to insulin signaling [87,88,89].

In Eastern populations, including those in Asia and the Arab region, distinct dietary patterns have been associated with distinct epigenetic signatures. Epigenome-wide association studies (EWAS) conducted in Qatari and Saudi cohorts have identified both shared and population-specific DNA methylation sites associated with diabetes. For example, an EWAS in an Arab cohort identified differential methylation at genes such as DQX1 and SOCS3, which were strongly correlated with glycemic traits, suggesting that region-specific dietary and environmental exposures shape the epigenetic architecture of T2D [90]. *SOCS3* (Suppressor of Cytokine Signaling 3) is a known mediator of insulin resistance and inflammation [91]; altered methylation of its promoter in Arab diabetics may help explain the heightened inflammatory profile observed in Middle Eastern patients with T2D [91]. While some epigenetic loci overlapped with those identified in Western EWAS, reflecting common pathways such as β-cell stress, others appeared to be unique to local environmental or genetic contexts.

Dietary differences could be a major contributor. High consumption of dates, a staple of Gulf diets, may induce specific epigenetic effects via polyphenols that are not commonly present in Western diets [92]. Conversely, high fructose intake from sugar-sweetened beverages in Gulf countries could uniquely methylate genes involved in fructose metabolism [93]. Another example is Ramadan fasting, a practice primarily observed by Muslim communities that involves a month of daytime fasting. Observational studies have reported transient metabolic and microbiome changes during Ramadan; however, direct evidence linking Ramadan fasting to sustained epigenetic modifications in metabolic tissues remains limited and requires further investigation [94].

Although several epigenetic pathways implicated in T2D appear broadly conserved across populations, the relative contribution of specific loci and environmental exposures may vary by dietary context and sociocultural environment. These differences warrant careful interpretation when extrapolating findings between Arab/MENA and Western cohorts.

## 10. Limitations and Research Gaps in Cross-Cultural Diet–Microbiome–Epigenome Studies

### 10.1. Methodological Limitations and Sources of Bias

Most evidence linking diet, microbiome features, and epigenetic marks in T2D remains observational and cross-sectional, limiting causal inference and increasing vulnerability to residual confounding [4,27,28,31]. Key sources of heterogeneity include differences in dietary assessment instruments, microbiome profiling methods (e.g., 16S rRNA versus shotgun sequencing and associated pipelines), and the tissue specificity of epigenetic measurements, with many studies relying on blood-based EWAS as biomarkers rather than direct readouts from metabolic tissues [4,27,31]. Medication exposure—particularly metformin—can influence epigenetic regulation and therefore requires careful adjustment or stratified analyses in cross-cohort comparisons [60]. Antibiotic exposure, smoking, adiposity/BMI, and physical activity can also affect metabolic and inflammatory phenotypes and should be consistently captured and addressed analytically.

In Arab and wider MENA settings, human data on diet–epigenome interactions in T2D remain sparse: most mechanistic insight is extrapolated from studies conducted in predominantly non-Arab populations or from experimental models of diet–epigenome crosstalk [28,31,45,52,85,86], with only a few population-based epigenetic investigations reported to date in Arab cohorts [90]. In the Gulf region, where T2D prevalence ranks among the highest globally [1,6,9,10,11,15,16,20], there are very few longitudinal studies that simultaneously monitor dietary patterns alongside multi-omics profiles—such as methylome, microbiome, and metabolome measures—limiting inferences about temporal and causal relationships.

To provide a comparative snapshot (not an exhaustive global prevalence summary), representative human studies reporting gut microbiome alterations associated with T2D across Arab/MENA and Western cohorts are summarized in Table 3.

**Figure 2:**
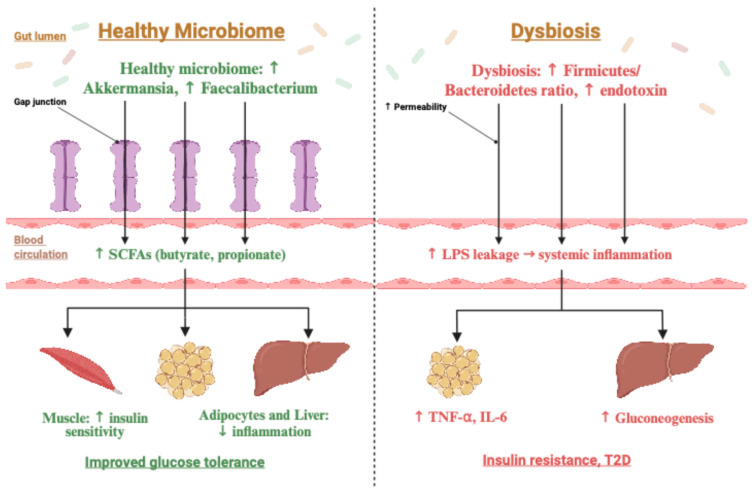
Comparative illustration of healthy (eubiotic) versus dysbiotic gut microbiome states and their metabolic consequences. The healthy microbiome (**left panel**) features increased *Akkermansia* and *Faecalibacterium*, which maintain tight junctions, support intestinal barrier integrity, and generate SCFAs such as butyrate and propionate. These metabolites reduce inflammation in adipose tissue and the liver, enhance muscle insulin sensitivity, and promote improved glucose tolerance. In dysbiosis (**right panel**), an elevated *Firmicutes*/*Bacteroidetes* ratio and increased endotoxin production impair barrier function, allowing LPS translocation and driving chronic inflammation. Elevated TNF-α and IL-6, along with hepatic gluconeogenesis, contribute to insulin resistance and T2D. The figure highlights intestinal barrier disruption as a key mechanistic link between microbiome composition and metabolic dysfunction.

Ideally, large-scale cohort studies in the Middle East should systematically collect detailed dietary information distinguishing between traditional and Westernized eating patterns and repeatedly measure epigenetic marks, gut microbiome composition, and metabolite profiles over time. Following participants from the prediabetic stage through diabetes onset would allow researchers to identify which dietary exposures most strongly predict epigenetic changes and future diabetes risk in Arab populations. For example, does a diet rich in dates and fiber, compared with one high in sugary beverages and saturated fats, produce distinct DNA methylation trajectories in young individuals who later develop T2D? Such studies could provide evidence for the effectiveness of culturally tailored dietary interventions in reversing adverse epigenetic changes. A conceptual overview of healthy versus dysbiotic gut microbiome states and their metabolic consequences relevant to T2D is shown in Figure 2 [23,54].

### 10.2. Arab/MENA-Specific Evidence Gaps

Arab/MENA populations remain underrepresented in large multi-omics T2D resources despite high disease burden. Key gaps include limited longitudinal cohorts with repeated dietary measurements and parallel profiling of the microbiome, metabolome, and epigenetic marks; insufficiently standardized metadata (dietary instruments, medication exposure, including metformin and antibiotics, and physical activity); and limited intervention trials testing culturally tailored dietary or lifestyle strategies with multi-omics endpoints.

### 10.3. Cross-Cultural Comparability and Western Cohort Heterogeneity

Cross-cultural inference is complicated by heterogeneity within Western cohorts (e.g., North American ultra-processed food exposure versus diverse European patterns including Mediterranean or Nordic diets) and by differences in baseline microbiome ecology across populations. Harmonized study designs, shared analytic pipelines, and comparable dietary classification frameworks are therefore critical to avoid overgeneralization when translating biomarkers or mechanistic interpretations across settings.

## 11. Role in Glucose Homeostasis and Insulin Resistance

The human gut microbiome, comprising trillions of microbes residing in the intestinal tract, has emerged as a key regulator of metabolic diseases, including T2D (Figure 2). These microbes function like a “hidden endocrine organ,” influencing nutrient absorption, energy harvest, immune signaling, and metabolic homeostasis. In healthy individuals, a diverse microbiota ferments dietary fiber into metabolites that enhance insulin sensitivity and modulate appetite [96]. In contrast, dysbiosis, an imbalance in microbial composition, can contribute to obesity and insulin resistance.

Several mechanisms link gut microbes to glucose homeostasis. First, microbiota influences the energy extracted from food. An excess of certain bacteria can increase caloric intake, predisposing individuals to gain weight [97]. Second, gut microbes produce metabolites that interact with host receptors. A key example is the short-chain fatty acid (SCFA) family, including butyrate, acetate, and propionate. Short-chain fatty acids (SCFAs) produced by microbial fermentation interact with host G-protein–coupled receptors and have been associated with incretin release (GLP-1, PYY) and improved insulin action [98,99,100]. Reduced SCFA production in dysbiosis may therefore attenuate these metabolic signals. SCFAs also directly improve insulin action in muscle and liver [100]. When fiber-fermenting bacteria are depleted, SCFA production declines, impairing these beneficial metabolic signals. Third, gut microbes regulate intestinal barrier integrity and inflammation [101]. Dysbiosis, often driven by high-fat diets, increases gut permeability, thereby allowing endotoxins, such as lipopolysaccharide (LPS), to enter circulation [102], triggering systemic inflammation and insulin resistance. Low-grade inflammation is a cornerstone of T2D pathophysiology, and the gut is a significant source of this inflammatory tone. Beneficial species such as *Akkermansia muciniphila* help maintain the mucus layer and prevent endotoxin leakage; depletion of these organisms is associated with metabolic inflammation [103]. Fourth, microbiomes influence bile acid metabolism. Gut bacteria convert primary bile acids into secondary forms that activate host receptors such as FXR and TGR5, thereby affecting glucose and lipid metabolism [104,105].

Overall, a eubiotic (healthy) microbiome promotes glycemic stability through multiple synergistic mechanisms: enhanced incretin release, improved gut barrier function, reduced inflammation, modulation of appetite, and regulation of adiposity [106,107].

In contrast, a diabetogenic microbiome, typically characterized by lower microbial diversity and increased abundance of detrimental species, tilts the metabolic balance toward insulin resistance and hyperglycemia [106]. Numerous studies have shown that individuals with T2D harbor an altered microbial composition compared with non-diabetic individuals, even independent of obesity [108]. Reduced microbial diversity, depletion of butyrate-producing bacteria, and expansion of opportunistic pathogens are consistent features of the T2D gut microbiome [95]. These changes may reflect bidirectional relationships with metabolic dysfunction, creating a feedback loop that perpetuates hyperglycemia.

## 12. Microbial Metabolites as Epigenetic Modulators

A key point of convergence between the gut microbiome and the host epigenome is the influence of microbial metabolites on epigenetic regulation. Metabolites produced by gut microorganisms can function as epigenetic modulators, thereby linking diet, microbial activity, and gene expression in host tissues. Short-chain fatty acids (SCFAs), discussed earlier as diet- and microbiome-derived epigenetic modulators, represent a central mechanistic link between microbial metabolism and host chromatin regulation. Rather than reiterating their basic biology, we focus here on their integrative role within multi-organ metabolic signaling networks relevant to T2D.

At the molecular level, SCFAs such as butyrate and propionate function as histone deacetylase (HDAC) inhibitors, thereby modulating histone acetylation and downstream gene expression programs involved in metabolic and inflammatory regulation [109,110,111,112]. These data provide direct mechanistic evidence that microbiome-derived metabolites can influence host chromatin structure and transcriptional activity.

Beyond SCFAs, bile acids modified by gut bacteria, including lithocholic acid (LCA) and deoxycholic acid (DCA), can bind to nuclear receptors such as FXR and PXR, thereby influencing the transcription of metabolic genes and functioning as key signaling molecules [113,114,115]. Microbial metabolism of dietary choline also generates trimethylamine N-oxide (TMAO), a compound linked to insulin resistance and atherosclerosis [116,117]. Evidence suggests that TMAO may alter DNA methylation patterns in macrophages, promoting pro-atherogenic gene expression [118].

Additionally, certain gut bacteria can synthesize vitamins (including biotin/vitamin B7 and vitamin B12) and polyamines that serve as cofactors for DNA methylation reactions or influence chromatin structure; biotin production has been reported for taxa including *Bacteroides*, *Escherichia coli*, *Bifidobacterium*, and *Lactobacillus* species [119,120]. Collectively, these findings highlight that the gut microbiota produces a variety of small molecules that can enter the bloodstream and reach distant organs, including the liver, adipose tissue, pancreas, and even the brain. These metabolites interact with host receptors or directly modulate epigenetic enzymes, altering gene expression and influencing glucose metabolism and overall metabolic health.

This represents a form of inter-kingdom communication in which microbial processing of dietary components shapes the signals sent to the host and ultimately influences the epigenome and metabolic phenotype. It mechanistically explains how changes in diet and microbiome compositions, such as the shift from traditional to Western dietary patterns, can become embedded in host cellular function through epigenetic pathways.

## 13. Comparative Microbiome Profiles: Arab vs. Western

The question of whether Arab populations exhibit gut microbiome configurations that differ from those reported in predominantly Western cohorts, and how such differences relate to T2D, remains largely open. Most large-scale microbiome–T2D associations to date have been characterized in non-Arab populations [95,106,108], whereas molecular studies in Arab cohorts have instead highlighted ancestry-specific epigenetic and metabolic signatures related to T2D and BMI [90]. These observations underscore the need for dedicated, harmonized microbiome studies in Gulf and broader Arab settings, and for cross-cohort designs that enable direct comparison with Western populations. In urban Western cohorts with T2D, a consistent finding is reduced abundance of short-chain fatty acid–producing bacteria. Multiple studies in Europe and the United States have reported significant depletion of *Faecalibacterium prausnitzii* and *Roseburia intestinalis*, both key butyrate producers, in individuals with T2D [121].

In contrast, some taxa appear more abundant in Middle Eastern guts. For example, *Prevotella*, a genus that thrives on fiber and participates in carbohydrate fermentation, is often reported at higher levels in populations consuming Arab/Mediterranean-style diets compared with those consuming Western diets [122,123]. A predominance of *Prevotella* has been linked to improved glycemic responses when dietary fiber intake is high [124]. However, in modern Arab diets with an elevated intake of simple sugars, *Prevotella* may also produce excess propionate, which has been associated with insulin resistance [125]. Preliminary evidence further suggests that Gulf Arab populations may exhibit a microbial functional shift toward amino acid degradation, including branched-chain amino acid (BCAA) metabolism, alongside reduced butyrate-synthesis potential, which could contribute to a higher metabolic risk [126].

Confirmatory metagenomic comparisons with Western cohorts remain necessary. Regarding overall diversity, some studies suggest that rural or traditional-diet Arab groups (e.g., Bedouins) harbor higher microbial diversity than urban Arabs, whose diets increasingly resemble those of Western cities [127]. Western urban populations typically exhibit reduced microbial diversity due to low-fiber, highly processed diets. Notably, similar reductions in diversity may now emerge among Gulf Arab populations as Westernized dietary patterns become more common.

Another relevant factor is the widespread use of herbal remedies and spices in Middle Eastern cuisine. Many of these contain antimicrobial or prebiotic compounds that may shape the microbiome differently than typical Western diets. For example, fenugreek, frequently used in the region, may selectively inhibit or promote gut microbes [128,129].

Although robust direct comparisons remain limited, current evidence supports the hypothesis that an Arab individual with T2D may possess a hybrid microbiome characterized by: (a) persistence of some fiber-responsive microbes, such as *Prevotella* (particularly if traditional dietary elements remain), and (b) an overlay of Western-diet effects, including loss of key butyrate producers and expansion of bacteria adapted to high fat and protein intake. Such a hybrid microbiome could generate metabolite profiles that intensify insulin resistance by combining adverse features of both dietary patterns. More comparative studies are needed, but acknowledging these differences is crucial for designing microbiome-based interventions tailored to each population.

## 14. How Diet Alters Microbiome Composition

Diet is a major determinant of gut microbiome composition, and changes in dietary intake can rapidly reshape microbial structure and function. High-fiber diets increase microbial diversity and beneficial taxa [130,131]. Additionally, Mediterranean-style dietary patterns enrich *Akkermansia muciniphila* and SCFA-producing microbes, which correlate with lower HbA1c and improved insulin sensitivity [132]. Traditional fermented foods introduce probiotic strains, such as Lactobacillus and Bifidobacterium, which support anti-inflammatory profiles [133].

In contrast, high-fat, low-fiber diets can alter microbiome composition within 24 h [134]. Ramadan fasting has been associated with transient increases in microbial richness and beneficial metabolites [135,136], including butyrate-producing taxa, although these shifts may reverse after resumption of prior habits [137].

Plant-based diets are linked to higher *Prevotella/Bacteroides* ratios, increased SCFA production, and reduced trimethylamine levels, a precursor of TMAO [138].

For Arab populations, emphasizing traditional plant-based staples and fermented dairy while limiting processed foods may help restore metabolically favorable microbiome configurations. Because baseline microbiome composition differs across populations, responses to dietary change may vary, highlighting the need for culturally tailored strategies.

## 15. Microbiome-Based Interventions

The growing awareness of the microbiome’s influence on metabolism has sparked interest in microbiome-targeted therapies for T2D. Several strategies are currently being explored. Probiotics, which introduce beneficial microbes, and prebiotics, which provide fermentable fibers that nourish these microbes, are among the most practical and widely studied approaches. Meta-analyses have reported modest but consistent benefits, indicating that probiotic supplementation may slightly reduce fasting blood glucose and *HbA1c* levels, likely by strengthening the gut barrier, reducing endotoxemia, and mitigating systemic inflammation [139]. However, results remain variable, partly because not all strains are effective for all individuals. Synbiotics, which combine probiotics with prebiotic fibers, have demonstrated slightly greater improvements in glycemic control than either component alone, as the fibers enhance probiotic survival and activity within the gut [140].

While probiotics, prebiotics, and synbiotics show modest average improvements in glycemic endpoints, effects vary substantially across strains, doses, baseline diets, and host characteristics. Many trials are limited by short duration, heterogeneous formulations, and inconsistent methods for microbiome profiling. Fecal microbiota transplantation (FMT), the transfer of stool from a metabolically healthy donor to a recipient to restore microbial ecology, has been explored in small proof-of-concept studies. Early European trials demonstrated transient improvements in insulin sensitivity after transfer of lean donor tissue to individuals with metabolic syndrome [141]. However, the durability of effect, optimal donor selection, long-term safety, regulatory oversight, and reproducibility remain unresolved. Benefits may also depend on sustained dietary modification to support microbial engraftment. At present, FMT cannot be considered an established therapeutic strategy for T2D and should be interpreted as a promising but investigational approach.

Enhancing the production of beneficial metabolites is another therapeutic strategy. It is increasingly clear that “one-size-fits-all” probiotics are not a universal solution; efficacy likely depends on an individual’s baseline microbiome composition. A strain that colonizes a European gut may not colonize an Arab gut with the same efficiency if ecological niches differ. The concept of precision probiotics has been proposed, wherein microbial supplementation strategies are tailored to baseline microbiome composition and dietary context. While biologically plausible, this approach remains largely theoretical, and robust clinical validation across diverse populations remains required.

A proof of concept for microbiome-targeted therapy has been demonstrated in fecal microbiota transplantation (FMT), in which the transfer of microbiota from lean donors improved insulin sensitivity in individuals with metabolic syndrome [142]. However, variability in clinical response underscores the importance of rational donor selection and ecological compatibility, supporting the need for precision microbiome-based interventions [143].

Microbiome-based interventions are being actively investigated as adjunct strategies for glycemic control; however, current evidence remains heterogeneous, and effect sizes are generally modest in clinical trials. A probiotic alone may have a limited impact without supporting dietary changes, whereas nutritional improvements can naturally enrich beneficial microbes even without supplementation. For Arab populations, combining traditional nutritional components rich in natural prebiotics with targeted probiotic supplementation may provide culturally aligned and effective therapeutic options. More broadly, these approaches highlight the importance of recognizing the microbiome as both a partner and a therapeutic target in T2D management, shifting toward an integrated host–microbe model rather than one focused solely on human biology.

## 16. Integrative Multi-Omics and Precision Medicine

The intricate connections among diet, the microbiome, and the epigenome in T2D highlight the need for an integrative multi-omics framework. Examining only a single layer, whether genetic, microbial, or epigenetic, provides an incomplete understanding of disease mechanisms. Integrating information from genomics, epigenomics, transcriptomics, metabolomics, and metagenomics offers a system-level view of how these biological networks interact to influence diabetes risk and progression. Multi-omic analyses have provided integrative insights into host–microbiome interactions; however, most findings remain associative and require validation in longitudinal and interventional settings. For instance, a recent study showed that the therapeutic effects of metformin involve coordinated regulation of both the gut microbiota and hepatic DNA-methylation patterns, linking microbial composition and metabolite shifts to synchronized epigenetic remodeling in the liver [144]. Similarly, Müller et al. (2024) performed a multi-omic integration of microbiome, methylome, and metabolome datasets, revealing that a pro-inflammatory gut microbiota profile is associated with altered hepatic DNA methylation signatures and elevated circulating branched-chain amino acids, these findings connect microbial dysbiosis to host epigenetic remodeling and metabolic imbalance [145].

Ensuring that Arab populations are adequately represented in multi-omics studies is essential to avoid Eurocentric biases in emerging models of diabetes. Most large multi-omics T2D cohorts have been conducted in Europe, the United States, or East Asia [146,147]. Their findings may not fully apply to Middle Eastern populations, which differ in genetic background (including region-specific variants and high consanguinity rates), predominant dietary patterns, and baseline gut microbiome composition. Underrepresentation of Arab populations in large multi-omics datasets may limit the generalizability of biomarker models and risk stratification tools derived predominantly from Western cohorts.

Overall, multi-omics integration offers a framework for linking dietary exposures, microbial function, and host gene regulation to clinically relevant T2D phenotypes. Nevertheless, translation into precision medicine requires standardized study designs, replication across diverse populations, and careful evaluation of cost-effectiveness and feasibility.

## 17. Conclusions and Applications

This review synthesizes evidence that diet–microbiome interactions can influence metabolic inflammation and insulin regulation through epigenetic mechanisms, providing a plausible molecular bridge between environmental exposures and T2D susceptibility. While much mechanistic and multi-omics evidence originates from Western cohorts, available data from Arab populations indicate potentially distinct dietary exposures, cultural practices, and baseline microbiome features that may modify these pathways, supporting the need for population-aware interpretation. From an application perspective, integrating dietary assessment with microbiome- and epigenome-informed biomarkers could support more targeted prevention strategies, but translation requires rigorous validation across diverse populations. Priorities include harmonized Arab-region cohorts with standardized diet/medication metadata (e.g., metformin), longitudinal sampling, and well-designed dietary or lifestyle intervention trials that can distinguish association from causation.

## Figures and Tables

**Table 1:** Epigenome-wide and candidate-gene methylation studies relevant to T2D in Arab and comparator populations. This table summarizes key findings from selected human studies and their DNA Methylation Signatures in T2D, directly related to the discussion of epigenetics and T2D.

Study (Population)	Sample & Assay	Key Methylation Findings (Loci & Direction)	Covariate Adjustment & Replication	Ref.
T2D patients vs. controls (Egyptian population)	~50 T2D vs. ~50 controls; bisulfite pyrosequencing of IGFBP1	Six CpG sites in IGFBP1 significantly hypermethylated in T2D (mean 30.6% vs. 22.8%, *p* ≈ 0.008)	Age- and sex-matched, single-gene study, no external replication	[45]
Meta-analysis of 5 prospective European cohorts	1250 incident T2D vs. 1950 controls; EWAS	76 CpGs associated with incident T2D (including TXNIP, ABCG1, SREBF1, CPT1A); many attenuated after BMI adjustment	Adjusted for age, sex, cell counts, prospective Cox models, replicated across cohorts	[46]
LOLIPOP cohort (Indian Asians) with European replication	1608 Indian Asians; replication in 306 Europeans; 450 K array	Five CpGs (ABCG1, PHOSPHO1, SOCS3, SREBF1, TXNIP) associated with future T2D; 5-CpG risk score predicted 8-year incidence (HR~3.5)	Age/sex matched, adjusted for metabolic factors, replicated in Europeans	[47]
Family-based EWAS (Mexican-American pedigrees)	850 individuals; family-based design; 450 K array	53 CpGs associated with T2D liability and glycemic traits; strong signals at TXNIP and ABCG1; ~7.8% variance explained	Adjusted for age, sex, familial relatedness, and internal family replication; meQTL analysis performed	[48]
EWAS in T2D and sustained hyperglycemia (Spain)	355 discovery + replication cohorts; 450 K array	cg19693031 (TXNIP) strongly hypomethylated in T2D (*p* = 1.17 × 10^−12^); inversely correlated with HbA1c	Adjusted for age, sex, BMI, smoking, and hyperlipidemia, replicated in independent cohorts	[49]
Sub-Saharan African EWAS (RODAM study)	713 Ghanaians (256 T2D, 457 controls); 450 K array	Significant CpGs at TXNIP, C7orf50, CPT1A, and African-specific TPM4; TXNIP remained significant after BMI adjustment	Adjusted for age, sex, BMI, cell-type composition, batch, and overlap with European loci	[50]
KoGES Ansung–Ansan cohort (Korea)	247 T2D vs. 887 controls; 450 K array	106 DMPs including TXNIP, ABCG1, C7orf50; novel loci (PDK4, ARRDC4, UFM1); 62 DMRs identified	Adjusted for age and sex, overlap with Western loci supports cross-population consistency	[51]
Integrated multi-omics Middle Eastern cohort	Integrated epigenome, whole-genome sequencing, and metabolome analyses	Identified multi-omics pathways linking DNA methylation, genetic variation, and metabolic intermediates in T2D	Adjusted for age, sex, BMI, integrative pathway analysis, and Middle Eastern population	[52]
ARIC study (Black and White Americans)	3120 adults; ~17-year follow-up; 450 K array	Seven novel CpGs (MICOS10, ZNF2, JPH3, GPX6); replicated known loci (TXNIP, CPT1A, ABCG1); DMRs in ADCY7 and TP63	Adjusted for age, sex, study center, cell composition, captures shared and ethnicity-specific markers	[53]

**Table 2:** Dietary components and their epigenetic mechanisms in metabolic regulation and T2D risk.

Dietary Component/Pattern	Epigenetic Mechanism	Human Evidence	Relevance to T2D/Metabolism	Refs.
Gut microbiota alterations in T2D	Altered microbial composition and metabolic functional potential	Systematic review summarizing consistent gut dysbiosis patterns in T2D patients	Supports microbiome involvement in T2D pathophysiology	[71]
Functional gut microbiome profiles in T2D	Altered microbial functional pathways associated with metabolic phenotypes	A 2023 study demonstrating functional microbiome signatures with predictive capacity for T2D	Suggests microbiome-based metabolic risk stratification	[72]
Dietary fiber intake	Fiber-associated microbiota shifts and circulating metabolite profiles	Human cohort linking fiber intake to specific gut taxa and blood metabolites related to T2D risk	Strengthens diet–microbiome–metabolite axis in metabolic regulation	[73]
Saudi population gut microbiome profiles	Population-specific microbial composition differences	Saudi cohort demonstrating significant microbiota associations with T2D-related phenotypes	Provides Arab-specific microbiome evidence	[74]
Polyphenol-rich Green-Mediterranean diet	Broad DNA methylation remodeling	DIRECT PLUS RCT showing extensive DMR changes and enhanced epigenetic regulatory potential with Green-MED diet	Suggests dietary polyphenols modulate epigenetic regulation alongside metabolic improvement	[75]
Microbiome-derived SCFAs	HDAC inhibition; histone acetylation and crotonylation modulation; regulation of inflammatory gene expression	Mechanistic and translational evidence demonstrating SCFA-mediated chromatin regulation	Links microbial metabolites to inflammatory and metabolic pathways relevant to insulin resistance	[76,77]
Methylation-supportive diet pattern	Reduction in DNA methylation age	Secondary dietary analysis associating specific dietary patterns with lower epigenetic age	Indicates dietary influence on biological aging trajectories relevant to metabolic risk	[78]
Polyphenol intake (Green-MED)	DNA methylation age attenuation	DIRECT PLUS analysis showing reduced epigenetic age associated with higher polyphenol intake	Suggests diet influences biological aging relevant to T2D prevention and progression	[79]
Lifestyle intervention (diet + physical activity)	Slowing of DNA methylation-based aging	Finnish Diabetes Prevention Study showed decelerated epigenetic aging after 2-year intervention	Supports epigenetic mediation of diabetes prevention	[80,81]
Diet and lifestyle intervention (pilot RCT)	Epigenetic age reversal	Randomized trial demonstrated significant DNAm-age reduction after 8 weeks	Suggests short-term lifestyle modification alters epigenetic aging markers	[82]
Dietary fiber–microbiome–inflammation axis	Fiber-associated microbiome modulation influencing inflammatory processes	Review linking fiber intake, microbiome composition, and inflammatory disease	Provides mechanistic support for fiber-driven metabolic and immune regulation	[83]
Nutrition strategies & epigenetic clocks	DNA methylation remodeling across clinical trials	Systematic review of dietary interventions targeting DNAm-age and DNA methylation	Synthesizes evidence for diet-induced modification of epigenetic aging relevant to metabolic disease	[84]

The table above is illustrative and not intended to estimate prevalence; a fuller catalog is beyond the scope of this review. Laban is a traditional fermented milk drink widely consumed in the Middle East and North Africa, as well as in parts of Central and South Asia.

**Table 3:** Representative human studies reporting gut microbiome alterations associated with T2D across Arab/MENA and Western cohorts.

Region	Sample	Sequencing Method	Key Findings	Reference
Saudi Arabia	461 T2D vs. 119 controls	16S rRNA gene sequencing	Altered community structure with enrichment of *Firmicutes*-associated taxa; variability in the *Firmicutes*/*Bacteroidetes* ratio reported across cohorts.	[74]
UAE	40 T2D vs. 44 controls	Nanopore metagenomic sequencing	Enterotypes identified: *Bacteroides*, *Firmicutes*, *Prevotella*. T2D is enriched for amino-acid degradation pathways. T2D enriched for urea metabolism pathways, biomarkers: *Absidia* spp., *Eubacterium limosum*.	[72]
Multi-region human cohort	Multiple cohorts	Shotgun metagenomics + metabolomics	Dietary fibre increased SCFA-producing bacteria. SCFA metabolites linked to improved insulin sensitivity. Supports fibre → microbiome → SCFA → metabolic-health mechanism.	[73]
Netherlands	2166 individuals (193 T2D)	16S rRNA gene sequencing	Lower richness in T2D. Depletion of butyrate-producers (Christensenellaceae, Ruminococcaceae). These taxa are inversely associated with T2D.	[95]

## Data Availability

Not applicable. No new datasets were generated or analyzed in this review.

## Abbreviations

AGEs	Advanced Glycation End-Products
BCAA	Branched-Chain Amino Acids
BMI	Body Mass Index
DALYs	Disability-Adjusted Life Years
DCA	Deoxycholic Acid
DNAm	DNA Methylation
DNMT	DNA Methyltransferase
EWAS	Epigenome-Wide Association Study
FFAR2	Free Fatty Acid Receptor 2
FMT	Fecal Microbiota Transplantation
FXR	Farnesoid X Receptor
GBD	Global Burden of Disease
GLP-1	Glucagon-Like Peptide-1
GLP1R	Glucagon-Like Peptide-1 Receptor
GPCR	G-Protein-Coupled Receptor
HAT	Histone Acetyltransferase
HbA1c	Glycated Hemoglobin
HDAC	Histone Deacetylase
HOMA-IR	Homeostatic Model Assessment of Insulin Resistance
IDF	International Diabetes Federation
IGT	Impaired Glucose Tolerance
LCA	Lithocholic Acid
lncRNA	Long Non-Coding RNA
LPS	Lipopolysaccharide
MAFLD	Metabolic Dysfunction-Associated Fatty Liver Disease
MENA	Middle East and North Africa
miRNA	MicroRNA
ncRNA	Non-Coding RNA
PYY	Peptide YY
RCT	Randomized Controlled Trial
SCFAs	Short-Chain Fatty Acids
SES	Socioeconomic Status
SIRT1	Sirtuin 1
T2D	T2D
TGR5	Takeda G-Protein-Coupled Receptor 5
TMAO	Trimethylamine N-Oxide
TXNIP	Thioredoxin-Interacting Protein
